# Identifying climate refugia and its potential impact on Tibetan brown bear (*Ursus arctos pruinosus*) in Sanjiangyuan National Park, China

**DOI:** 10.1002/ece3.5780

**Published:** 2019-11-14

**Authors:** Yunchuan Dai, Charlotte E. Hacker, Yuguang Zhang, Wenwen Li, Yu Zhang, Haodong Liu, Jingjie Zhang, Yunrui Ji, Yadong Xue, Diqiang Li

**Affiliations:** ^1^ Research Institute of Forest Ecology, Environment and Protection Chinese Academy of Forestry Beijing China; ^2^ Key Laboratory of Biodiversity Conservation State Forestry and Grassland Administration Beijing China; ^3^ Department of Biological Sciences Duquesne University Pittsburgh PA USA; ^4^ Key Laboratory for Biodiversity Science and Ecological Engineering Ministry of Education College of Life Sciences Beijing Normal University Beijing China; ^5^ Qilian Mountain National Park Qinghai Administration Xining China; ^6^ Research Institute of Forest Resource Information Techniques Chinese Academy of Forestry Beijing China; ^7^ Key Laboratory of Adaptation and Evolution of Plateau Biota Northwest Institute of Plateau Biology Chinese Academy of Sciences Xining China

**Keywords:** Circuit model, climate refugia, corridor, habitat connectivity, *Ursus arctos pruinosus*

## Abstract

Climate change has direct impacts on wildlife and future biodiversity protection efforts. Vulnerability assessment and habitat connectivity analyses are necessary for drafting effective conservation strategies for threatened species such as the Tibetan brown bear (*Ursus arctos pruinosus*). We used the maximum entropy (MaxEnt) model to assess the current (1950–2000) and future (2041–2060) habitat suitability by combining bioclimatic and environmental variables, and identified potential climate refugia for Tibetan brown bears in Sanjiangyuan National Park, China. Next, we selected Circuit model to simulate potential migration paths based on current and future climatically suitable habitat. Results indicate a total area of potential suitable habitat under the current climate scenario of approximately 31,649.46 km^2^, of which 28,778.29 km^2^ would be unsuitable by the 2050s. Potentially suitable habitat under the future climate scenario was projected to cover an area of 23,738.6 km^2^. Climate refugia occupied 2,871.17 km^2^, primarily in the midwestern and northeastern regions of Yangtze River Zone, as well as the northern region of Yellow River Zone. The altitude of climate refugia ranged from 4,307 to 5,524 m, with 52.93% lying at altitudes between 4,300 and 4,600 m. Refugia were mainly distributed on bare rock, alpine steppe, and alpine meadow. Corridors linking areas of potentially suitable brown bear habitat and a substantial portion of paths with low‐resistance value were distributed in climate refugia. We recommend various actions to ameliorate the impact of climate change on brown bears, such as protecting climatically suitable habitat, establishing habitat corridors, restructuring conservation areas, and strengthening monitoring efforts.

## Graphical abstract

Determining regions of refugia and climate connectivity enable the identification of the most effective areas to maintain brown bear populations and enhance connectivity against the background of climate change projection in this century.

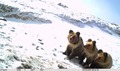

## 1 INTRODUCTION

The earth is undergoing rapid shifts in climate (IPCC, 2014), which will have severe effects on biodiversity. Understanding such impacts is a matter of urgency (Aryal, Brunton, & Raubenheimer, 2013; Garcia, Cabeza, Rahbek, & Araujo, 2014; Vegas‐Vilarrúbia, Nogué, & Rull, 2012). Strong evidence indicates that climate change has significant impacts on species' phenology (Cohen, Lajeunesse, & Rohr, 2018; Tomotani, Gienapp, Beersma, & Visser, 2016), behavior (Papaj, Mallory, & Heinz, 2007; Rockwell & Gormezano, 2008), distribution and richness (Aryal et al., 2016; Ihlow et al., 2012), population size and interspecies relationships (Cohen et al., 2018), and ecosystem structure and function (Cramer et al., 2001; Li, Li, Zhao, Zheng, & Bai, 2018), all of which exacerbate the rate of species extinction (Lewis, 2006; Mammola, Goodacre, & Isaia, 2017). The Intergovernmental Panel on Climate Change (IPCC) estimates that 20%–30% of species are facing extinction in this century if the global average temperature rises 2–3°C above preindustrial levels (IPCC, 2014).

Researchers have simulated models that minimize extinction risk by identifying species and habitats susceptible to climate change and how wildlife may respond to large scale environmental shifts (Balzotti, Kitchen, & McCarthy, 2016; Foden et al., 2013; Guisan et al., 2013). Species distribution models (SDMs) use environmental variables to explain both current and future distributions (Li et al., 2019; Struebig et al., 2015). SDMs have become essential for approaching research challenges in fields such as biogeography, evolution, ecology, and conservation biology (Guisan & Thuiller, 2005). At present, researchers have used the maximum entropy (MaxEnt) model to assess the habitat suitability for a variety of rare or endangered wildlife around the world (Bai et al., 2018; Li, Liu, Xue, Zhang, & Li, 2017; Li et al., 2019; Zhang, Jiang, et al., 2019; Zhang, Clauzel, et al., 2019). Furthermore, the Circuit model is commonly used by natural resource managers to predict wildlife dispersal paths and design ecological corridors, which are often used in wildlife management and conservation practice (Li et al., 2019; McRae & Beier, 2007; McRae, Shah, & Mohapatra, 2013; Walpole, Bowman, Murray, & Wilson, 2012; Zhang, Clauzel, et al., 2019).

Paleoecological records and observed species migrations indicate that species distributions follow suitable climatic conditions (Parmesan & Yohe, 2003). However, changes in distributions are limited by climate, landscape features, and dispersal potential (Lambers, 2015; Littlefield, McRae, Michalak, Lawler, & Carroll, 2017). Refugia are areas which possess relatively stable climatic conditions with high connectivity between suitable habitat in different climate scenarios (Littlefield et al., 2017; Morán‐Ordóñez, Briscoe, & Wintle, 2017). Knowledge of current and future habitat refugia of threatened species, such as the Tibetan brown bear (*Ursus arctos pruinosus*) is vital in designing conservation plans aimed at promoting long‐term species persistence.

**Figure 1:**
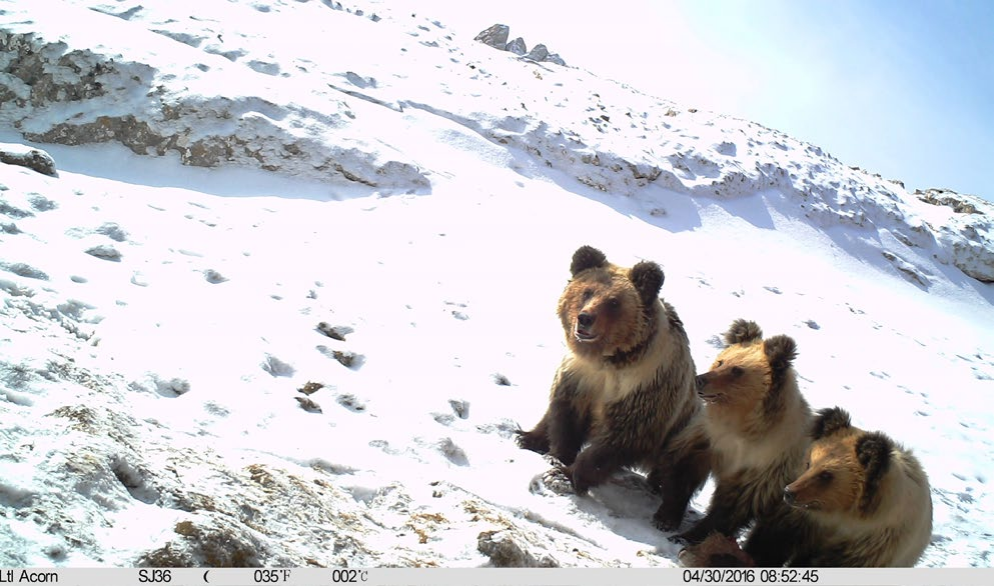
Tibetan brown bear (*Ursus arctos pruinosus*) captured by camera trapping in the Yangtze River Zone of Sanjiangyuan National Park, China

The Tibetan brown bear, also known as the Tibetan blue bear, is a rare brown bear subspecies living at high altitudes in close proximity to humans in Asia (Aryal et al., 2012; Aryal, Sathyakumar, & Schwartz, 2010; Xu et al., 2006; Figure 1). The species population estimate is 5,000–6,000 individuals (Wu, 2014). Sanjiangyuan National Park of China provides important habitat and migration corridors for the species. At present, their primary threat is habitat reduction and fragmentation (Aryal et al., 2010; Coulon et al., 2004; Littlefield et al., 2017; McRae & Beier, 2007). A habitat assessment for Tibetan brown bears by Wu (2014) used species distribution data and eco‐geographic variables, combined with Generalized Linear Models, to assess species‐appropriate habitat in the Suojia region of Sanjiangyuan National Park. However, this study did not consider climate change, leaving a substantial knowledge gap in our understanding of its potential impacts on species distribution.

This research constructed a projected distribution model for brown bears based on presence data and related bioclimatic and environmental factors. We used Circuit model to simulate the potential movement paths of brown bears under both current and future climate scenarios. The aims of this work were to (a) project current and future climatically suitable habitat for brown bears, (b) identify climate refugia, and (c) recognize dispersal paths that allow for migration from current to future suitable habitat. Our findings will be incorporated into a brown bear protection plan in the context of global climate change in Sanjiangyuan National Park, China.

## 2 MATERIAL AND METHODS

### 2.1 Study area

**Figure 2:**
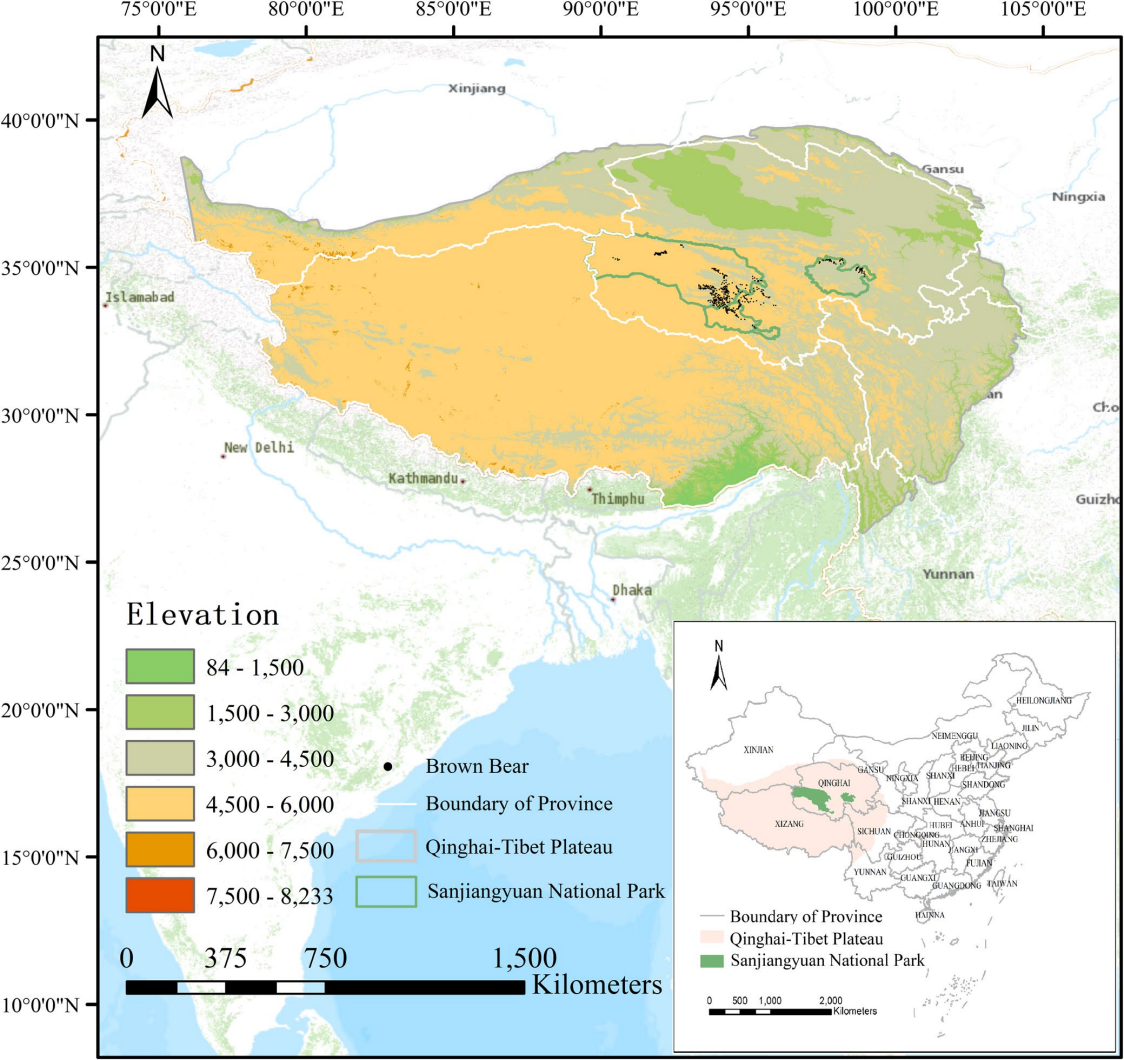
Location of Sanjiangyuan National Park, China

Sanjiangyuan National Park is China's first pilot national park. It lies in the hinterland of the Tibetan Plateau (between 89°50′ and 99°14′E, 32°22′ and 36°47′N), spanning an area of 123,100 km^2^, 14 times larger than Yellowstone National Park (Figure 2). The altitude is between 3,500 and 4,800 m. It is a plateau continental climate. The weather is typically dry and cold with the annual average temperature ranging from −5.6°C to −7.8°C and the annual precipitation consisting predominately of snowfall ranging from 262.2 mm to 772.8 mm. Sanjiangyuan, or Source of Three Rivers, refers to the area's role as the headwaters of China's three largest rivers (Yangtze river, Yellow river and Lancang river). The region has global influence and dictates China's ecosystem (Zhang, Jiang, et al., 2019). A variety of endemic alpine flora and fauna constitutes Sanjiangyuan's excessive biological diversity. As China's first national park, it has become an exhibition area of nature protection and ecological culture heritage on the Tibetan Plateau (Zhang, Jiang, et al., 2019). Unfortunately, it is also one of the most sensitive regions to climate change (Liu et al., 2017; Wang, Song, & Hu, 2010).

### 2.2 Data preparation

We collected 528 GPS coordinates of brown bear. Among them, 315 were obtained via ground surveys from 2016 to 2018 (recorded coordinates of brown bear presence, including feces, footprints, hair, and foraging traces), 65 from infrared camera traps and 148 from published literature (Wu, 2014; Xu et al., 2006; Figure 2). To reduce autocorrelation, presence points were filtered by randomly selecting one point in each 1 km^2^ grid (Aryal et al., 2016; Li et al., 2017; Zhang, Jiang, et al., 2019).

Land use/land cover data of the study area were obtained by interpreting 2017 Landsat 8 OLI (at 30 m resolution; U.S. Geological Survey; https://www.usgs.gov/), and adopting a 1:50,000 digital elevation model (DEM) as a reference control image to correct for geometric biases by using ENVI 5.1 (ESRI Inc.). An RMS error <1 indicates that the land use/land cover data fulfill the precision standards of research. Land use/land cover type was organized into 15 categories: (1) forest, (2) bush, (3) grassland, (4) alpine meadow, (5) alpine steppe, (6) irrigable land, (7) dry land, (8) construction land, (9) swamp, (10) river bed, (11) ice, (12) bare rock, (13) bare land, (14) desert, and (15) water body.

The 19 bioclimatic factors (at 1 km resolution) undercurrent (average for 1950–2000) and future climatic conditions (average for 2041–2060) were extracted from the WorldClim database (http://www.worldclim.org/version1). Future climate data consisted of IPCC and the Coupled Model Intercomparison Project 5 (CMIP5) climate projection (Wu, Chen, & Wen, 2013) from the Met Office Hadley Center for atmosphere‐ocean coupled climate model (HadGEM2‐AO) under the representative concentration pathways (RCPs) 4.5 (Baek et al., 2013). Under the RCP4.5 scenario, the global average temperature would rise by 0.9–2.0°C by the 2050s, consistent with the expectations of the Paris Agreement (UNFCCC, 2015). Previous studies (Choi, Lee, & Oh, 2011; Ahn et al., 2013; Baek et al., 2013; Hong & Ahn 2015) have found HadGEM2‐AO to have satisfactory performance in simulating climate change trends and general patterns of current climate over the East Asia region. In addition, previous studies have used HadGEM2‐AO to construct species distribution models under the future climate scenario in China (Li, Li, Xue, et al., 2018; Li et al., 2017, 2019). Therefore, we elected to use HadGEM2‐AO for predicting future distributions of Tibetan brown bear.

Additional environmental variables including altitude and Human Influence Index (HII) were combined to create the distribution model for brown bear. Altitude was derived from the ASTER GDEM V2 digital elevation model (at 30 m resolution; http://www.gscloud.cn/). HII was obtained from the Socioeconomic Data and Applications Center, NASA (Last of the Wild, v2; at 1 km resolution; http://sedac.ciesin.columbia.edu/). HII represents anthropogenic impacts (1995–2004), and was calculated by integrating human accessibility, human land use, and human population pressure. Nonclimatic factors for the 2050s would be unavailable; thus, these factors were kept static in prediction (Stanton, Pearson, Horning, Ersts, & Reşit Akçakaya, 2011).

All spatial variables (climate and nonclimate) were resampled to 1 km resolution and unified in a projection coordinate system (WGS_ 1984_UTM_Zone_47N) in ArcGIS 10.1 (ESRI Inc.). The correlation coefficients of variables were computed by using the tool of Band Collection Statistics (BCS) in ArcGIS 10.1. Variables were screened in a series of three steps to identify key variables affecting climatic suitability of brown bears. First, the multicollinearity of variables was reduced by eliminating correlation variables where |*r*| > 0.8 (Appendix 1; Cord, Klein, Mora, & Dech, 2014; Li et al., 2019). Second, the remaining variables were introduced to the model, and those with no contribution rates were removed. Third, the most influential variables based on contribution rates obtained from the output of the first model were selected, and the model repeated.

### 2.3 Habitat suitability model

MaxEnt model is considered one of the most efficient tools to predict species distribution with presence‐only data, leading to its widespread use (Aryal eT al., 2016; Gomes et al., 2018; Lamsal, Kumar, Aryal, & Atreya, 2018; Ma & Sun, 2018; Phillips, Anderson, & Schapire, 2006). The parameters of MaxEnt model were set to: 25% for random test percentage and 1 regularization multiplier. We ran 15 replicates and performed a cross validation (Phillips et al., 2006; Vedel‐Sørensen, Tovaranonte, Bøcher, Balslev, & Barfod, 2013). Percent contribution was used to estimate the importance of variables. The logistic results of MaxEnt were regarded as the probability of species occurrence, with values ranging from 0 to 1. A threshold value was used to distinguish between suitable and unsuitable regions. The average logistic threshold value of maximum training sensitivity plus specificity (MTSPS) was recommended (Liu, White, & Newell, 2013). Grids with probability values greater than the threshold were deemed suitable habitat. We then withdrew suitable patches with areas <10 km^2^ based on the known minimum home range of brown bears (Nagy & Haroldson, 1990).

We evaluated MaxEnt model performance by using the area under the receiver operating characteristic curve (AUC). AUC is an independent threshold value to verify the accuracy of model outputs. Values range from 0 to 1, with those closer to 1 indicating a more accurate model (Araujo, Pearson, Thuiller, & Erhard, 2005; Phillips et al., 2006).

### 2.4 Vulnerability assessment

Changes in potential suitable habitat under the current and future climate scenarios were assessed by identifying vulnerable habitat, increased suitable habitat and climate refugia. Their definitions are as follows:

1. Vulnerable habitat: Regions of habitat currently suitable and predicted to be unsuitable under the future climate scenario;
2. Increased suitable habitat: Regions of habitat currently unsuitable and predicted to be suitable under the future climate scenario;
3. Climate refugia: Regions of habitat currently suitable and predicted to be suitable under the future climate scenario.

Three indicators were selected to demonstrate the impacts of climate change on currently suitable brown habitat: (a) *AC*: suitable habitat change percentage; (b) *SH_c_*: current suitable habitat loss percentage; and (c) *SH_f_*: increased suitable habitat percentage under the future climate scenario (Duan, Kong, Huang, Varela, & Ji, 2016; Li et al., 2017). The formulas for each indicator are as follows:

AC=(Af-Ac)/Ac×100%

SHc=(Ac-Acf)/Ac×100%

SHf=(Af-Acf)/Af×100%

where *A_c_* is the projected area of current suitable habitat; *A_f_* is the projected area of future suitable habitat; and *A_cf_* is the area of climate refugia.

### 2.5 Geographical features of climate refugia

Altitude characteristics and typical land use types of climate refugia for brown bears were analyzed by overlaying the climate refugia map with the layers of land use types and altitude in ArcGis 10.1.

### 2.6 Habitat connectivity analysis

We simulated the potential migration paths for brown bears based on current and future habitat connectivity by using Circuit model (Circuitscape software 4.0; https://circuitscape.org/; Li et al., 2019; McRae & Beier, 2007; McRae et al., 2013; Walpole et al., 2012; Zhang, Clauzel, et al., 2019). The model mode, calculation, and mapping options for Circuitscape were set to: Pairwise mode (run in low‐memory mode), use average conductance instead of resistance for connections between cells, write cumulative and max current maps only, and set focal node currents to zero. We inverted the habitat suitable index (HSI) value to link the suitable habitat of brown bears with low movement resistance and vice versa. We used the functions of negative exponential transformation to convert HSI into resistance values (Keeley, Beier, & Gagnon, 2016):

If HSI>Threshold→Species Suitable Habitat→Resistance=1

$$\text{If HSI}<\text{Threshold}\to \text{Non}\;-\;\text{suitable Habitat}/\text{Matrix}\to \text{Resistance}=e^{\frac{\ln\left(0.001\right)}{\text{threshold}}\times \text{HSI}}\times 1000$$

## 3 RESULTS

### 3.1 Model performance

**Figure 3:**
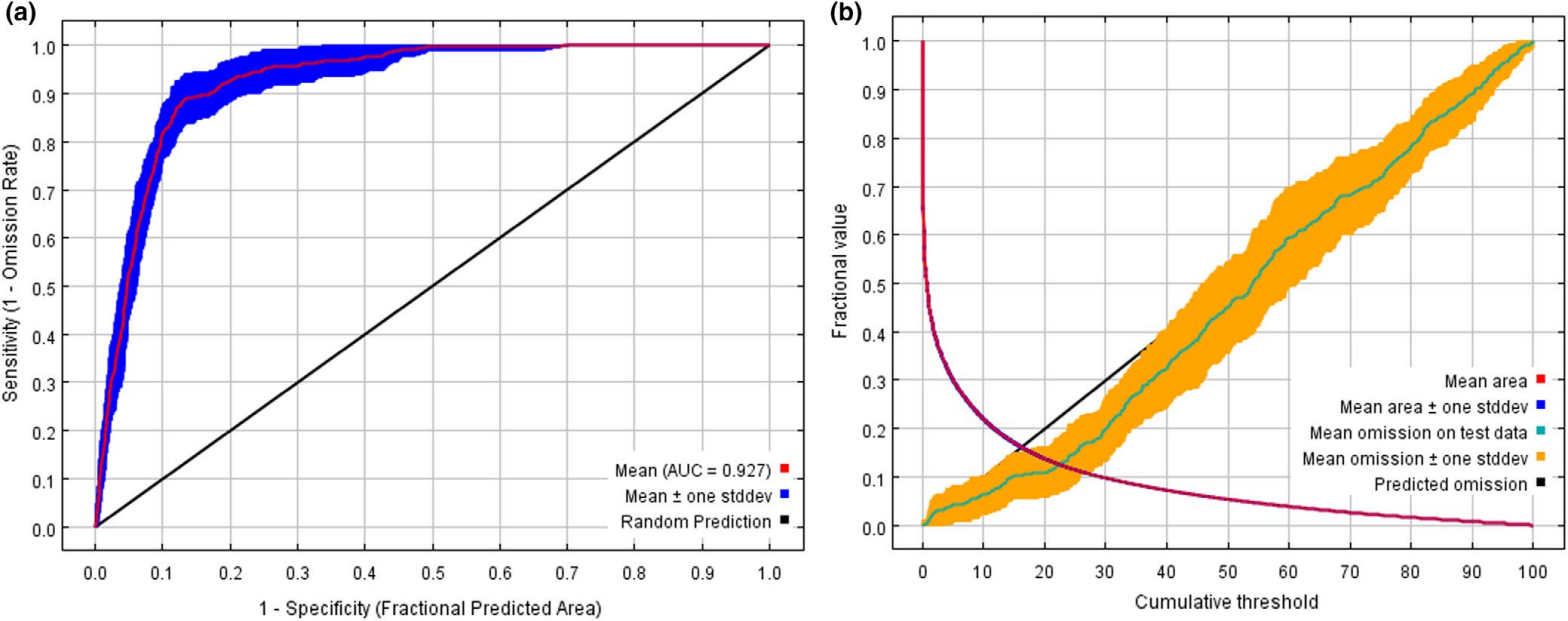
Statistical graphs of MaxEnt model output results. (a) the receiver operating characteristic (ROC) curve and average test AUC for accuracy analysis of habitat prediction by MaxEnt model, and (b) the analysis of test omission rate and predicted area, where values indicate the training gain only with variables

Distribution models were made with 387 GPS coordinates of brown bear and 7 variables to determine potentially suitable habitat. The percent contribution of model variables ranked from highest to lowest were as follows: Bio4 (Temperature Seasonality; 38.7%), Bio15 (Precipitation Seasonality; 27.7%), Bio3 (Temperature Constancy; 15.8%), Altitude (7.9%), Bio2 (Mean Diurnal Range; 5.5%), Bio14 (Precipitation of Driest Month; 3.7%), and HII (Human Influence Index; 0.7%) (Table 1). Cross validation illustrated sufficient performance for model outputs (average testing AUC was 0.9267 ± 0.0157; average training AUC was 0.936 ± 0.0007; Figure 3).

**Table 1:** Environmental factor definitions and their contribution rates

Code	Environmental factors	Unit	Contribution rate (%)
Bio1	Mean annual temperature	°C	
Bio2	Mean diurnal range	°C	5.5
Bio3	Temperature constancy	–	15.8
Bio4	Temperature seasonality (standard deviation *100)	–	38.7
Bio5	Max temperature of warmest month	°C	
Bio6	Min temperature of coldest month	°C	
Bio7	Temperature annual range (Bio5‐Bio6)	°C	
Bio8	Mean temperature of wettest quarter	°C	
Bio9	Mean temperature of driest quarter	°C	
Bio10	Mean temperature of warmest quarter	°C	
Bio11	Mean temperature of coldest quarter	°C	
Bio12	Annual precipitation	mm	
Bio13	Precipitation of wettest month	mm	
Bio14	Precipitation of driest month	mm	3.7
Bio15	Precipitation seasonality (Coefficient of variation)	–	27.7
Bio16	Precipitation of wettest quarter	mm	
Bio17	Precipitation of driest quarter	mm	
Bio18	Precipitation of warmest quarter	mm	
Bio19	Precipitation of coldest quarter	mm	
Altitude	Altitude	m	7.9
HII	Human Influence Index	–	0.7

### 3.2 Changes in potential suitable habitat

**Figure 4:**
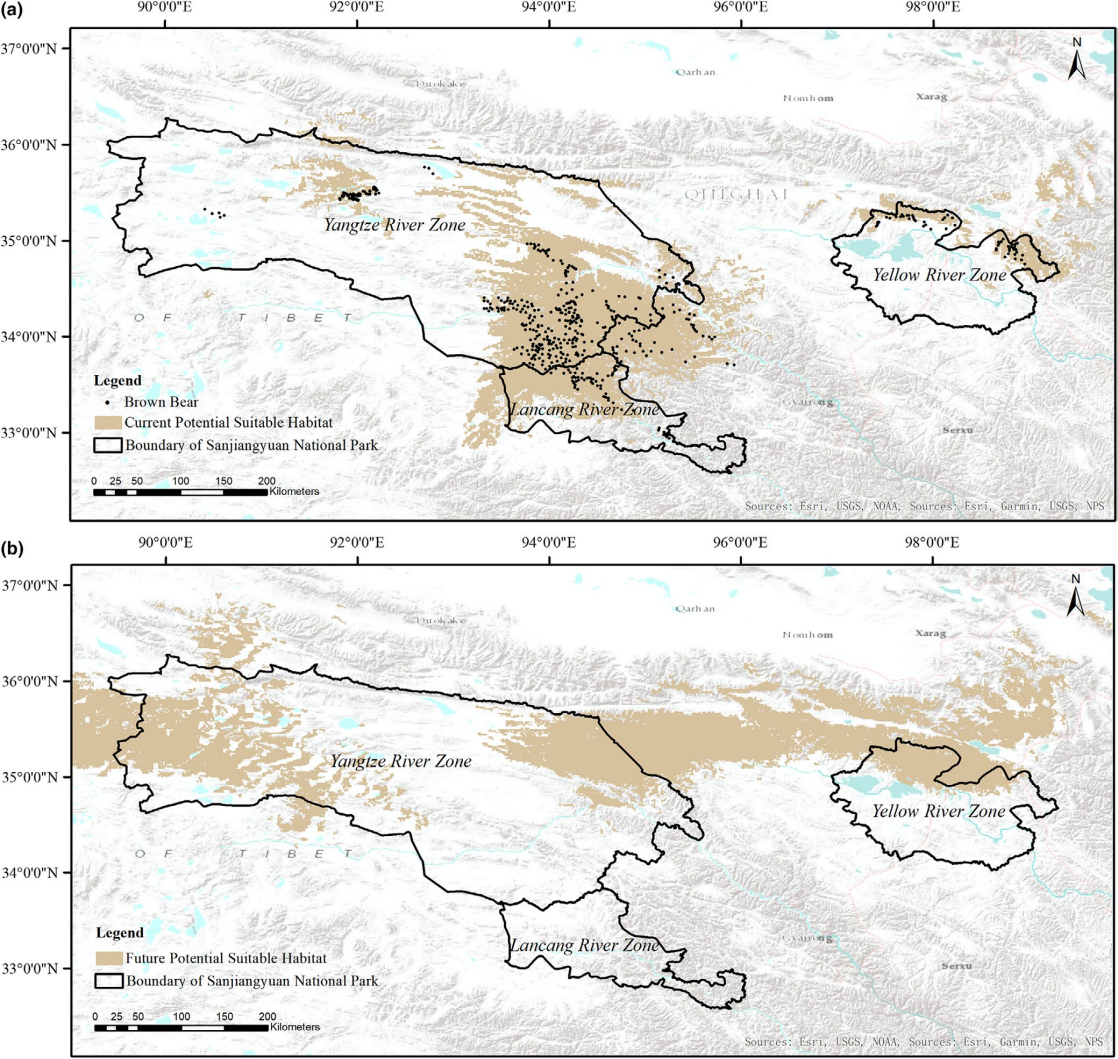
Potential suitable habitat of brown bear in Sanjiangyuan National Park. (a) represents the model outputs under the current climate scenario and (b) the prediction of suitable habitat under the future climate scenario

The average logistic threshold value of MTSPS was 0.3562. Cells with a value of habitat suitability <0.3562 covered an area of 31,649.46 km^2^ under the current climate scenario in Sanjiangyuan National Park (Table 2). Potential suitable habitat for brown bears was primarily distributed in the southeastern region of the Yangtze River Zone, northwestern region of the Lancang River Zone, and northern region of the Yellow River Zone (Figure 4a). Under the future climate scenario, the area of potential suitable habitat was projected to be 26,609.77 km^2^ (Table 2), a reduction of 5,039.69 km^2^. Habitat reduction primarily occurred in the Lancang River Zone, the southeastern region of the Yangtze River zone and the northeastern region of the Yellow River Zone (Figure 4b).

**Table 2:** Predicted changes of potential suitable habitat for brown bears in Sanjiangyuan National Park

Sanjiangyuan National Park	*A_c_* (km^2^)	*A_f_* (km^2^)	*A_cf_* (km^2^)	*AC* (%)	*SH_c_* (%)	*SH_f_* (%)
Yangtze River Zone	23,204.44	22,734.84	1,614.72	–2.02	93.04	92.90
Yellow River Zone	2,380.54	3,874.93	1,256.45	62.78	47.22	67.57
Lancang River Zone	6,064.48	0	0	–100.00	100.00	0
Total	31,649.46	26,609.77	2,871.17	–15.92	90.93	89.21

We found that potential suitable habitat of brown bears in all regions except the Yellow River Zone (*AC* = 62.78%) would decrease under the future climate scenario. In the 2050s, the potential suitable habitat area of brown bears in the Yangtze River Zone was reduced by 469.6 km^2^ (*AC *= −2.02%), and the potential suitable habitat area of brown bears in the Lancang River Zone reduced by 6,064.48 km^2^ (*AC* = −100%). Suitable habitat in the Yellow River Zone increased by 1,494.39 km^2^ (*AC* = 62.78%) under the future climate scenario (Table 2 and Figure 4b).

### 3.3 Vulnerability assessment

**Figure 5:**
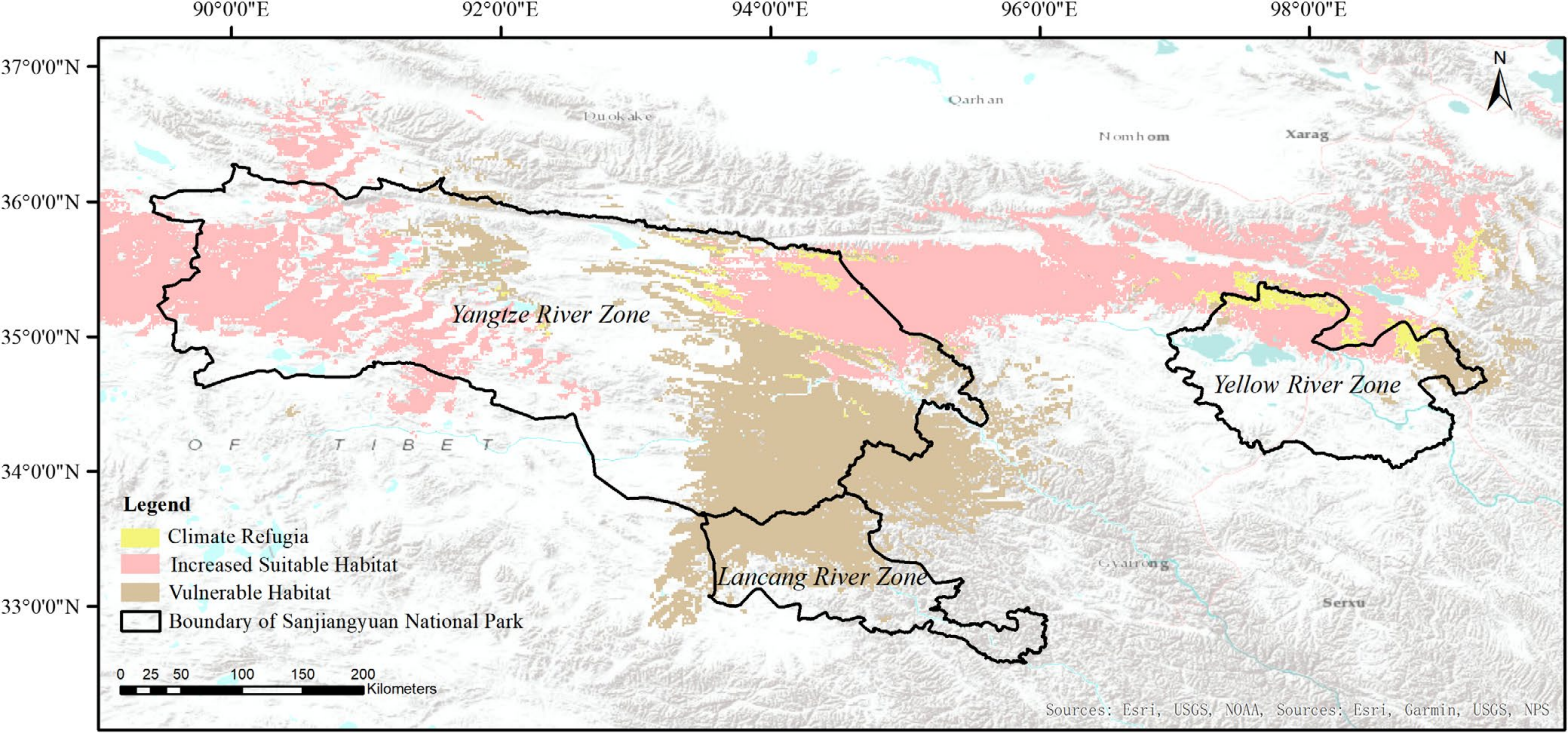
Vulnerability analysis of potential suitable brown bear habitat in Sanjiangyuan National Park

Due to climate change, 28,778.29 km^2^ (*SH_c_* = 90.93%) of current suitable brown bear habitat was predicted to be vulnerable. Areas of potential suitable habitat under the future climate scenario covered 23,738.6 km^2^ (*SH_f_* = 89.21%) and were mainly distributed in the northwestern and northeastern region of the Yangtze River Zone and northern region of the Yellow River Zone. The area of climate refugia was 2,871.17 km^2^, and aggregated in the midwestern and northeastern regions of the Yangtze River Zone, as well as the northern part of the Yellow River Zone (Table 2, Figure 5).

### 3.4 Geographical features of climate refugia

**Figure 6:**
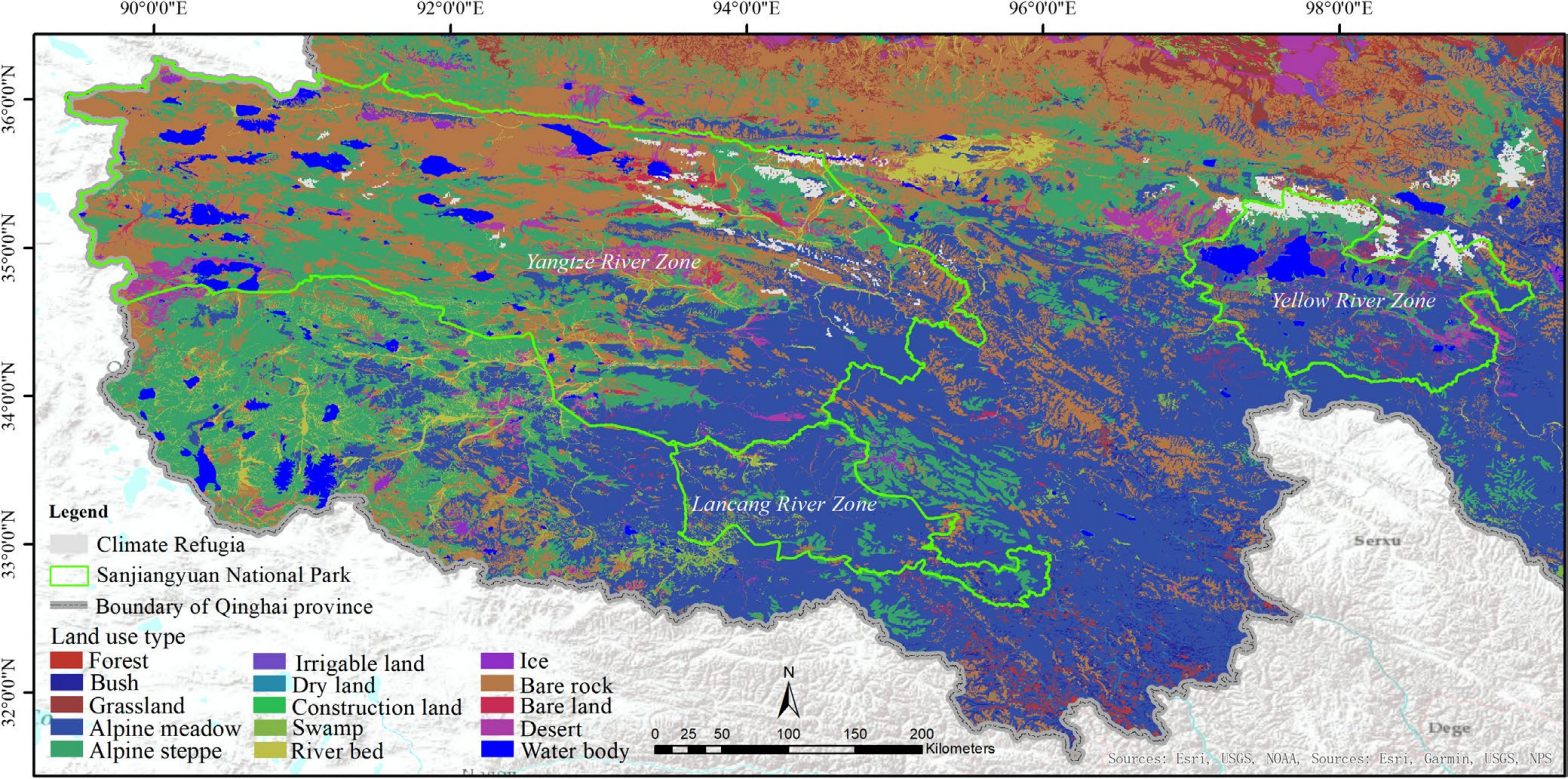
The land use types of climate refugia for brown bears in Sanjiangyuan National Park

Climate refugia ranged in altitude from 4,307 to 5,524 m, primarily falling between 4,300 and 4,600 m (1,519.74 km^2^; 52.93%) followed by 4,601–4,900 m (873.25 km^2^; 30.41%), 4,901‐5,200 m (458.46 km^2^; 15.97%), and >5,201 m (19.72 km^2^; 0.69%). Most climate refugia areas were distributed on bare rock (1,028.24 km^2^; 35.81%; Figure 6) followed by alpine steppe (829.64 km^2^; 28.90%), alpine meadow (744.38 km^2^; 25.93%), bare land (115.8 km^2^; 4.03%), swamp (76.11 km^2^; 2.65%), desert (33.1 km^2^; 1.15%), river bed (32.02 km^2^; 1.12%), ice (8.56 km^2^; 0.3%), and water body (3.32 km^2^; 0.12%).

### 3.5 Brown bears' potential movement paths

**Figure 7:**
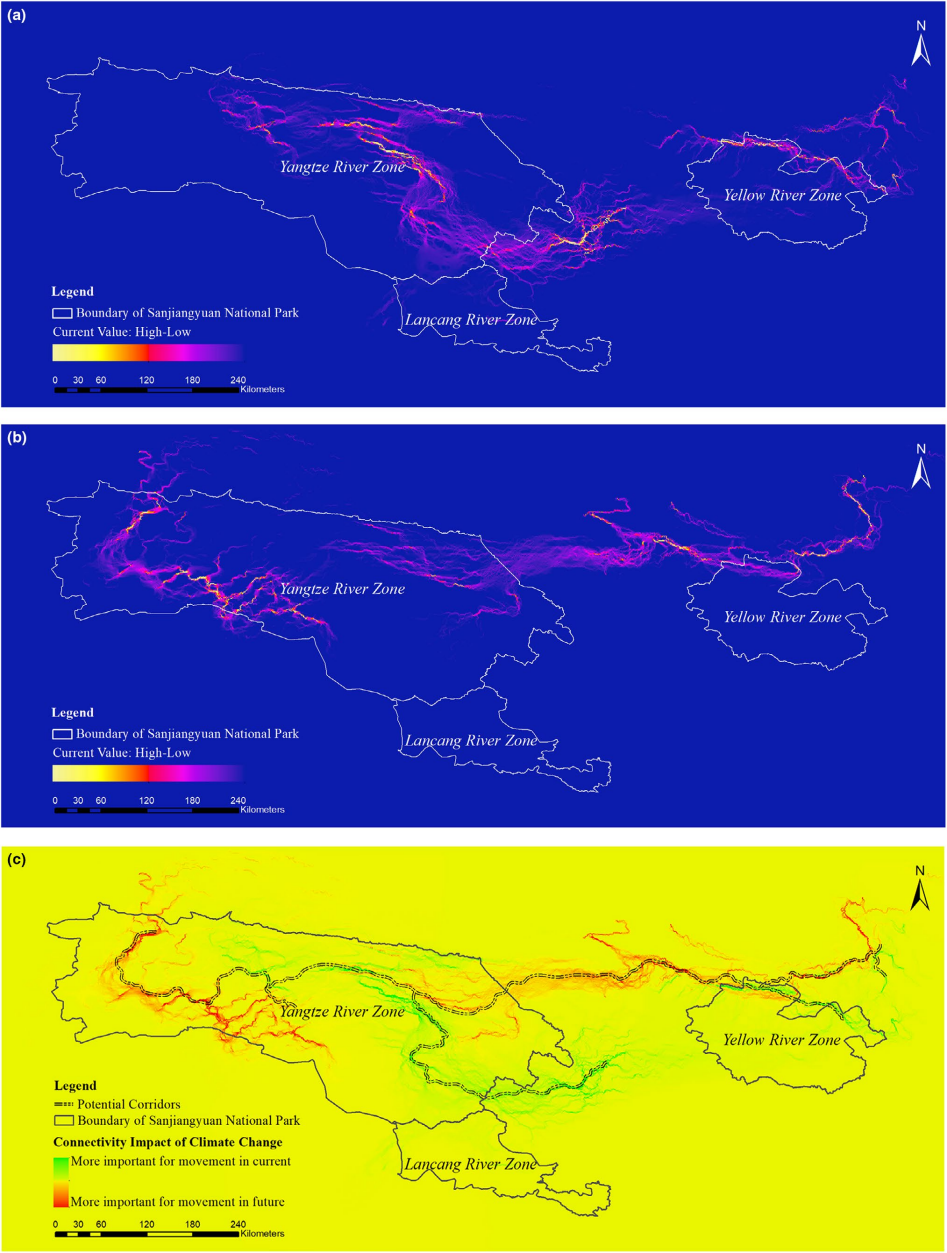
Potential movement paths for brown bears in Sanjiangyuan National Park simulated by the Circuit model based on current and future suitable habitat. (a) current connectivity and (b) future connectivity, and (c) impact of climate change on connectivity

The north central areas of the Yangtze River Zone and northern area of the Yellow River Zone exhibited high‐current flow undercurrent and future climate scenarios (Figure 7a,b). Under the current climate scenario, high‐current areas existed in the central of Lancang River Zone and the southeastern region of Yangtze River Zone, but these did not persist under the future climate scenario. Additional migration paths emerged in the northeastern and western regions of Yangtze River Zone under the future climate scenario. Potential migration routes for brown bears were primarily distributed in vulnerable habitat, whereas routes important for future movement were primary located in newly suitable habitat (Figure 7c).

## 4 DISCUSSION

The decline in habitat connectivity and quality has led to a highly fragmented distribution of Tibetan brown bears (Aryal et al., 2010; Nawaz, Martin, & Swenson, 2014). Vast areas of preferred brown bear habitat, alpine meadows and grasslands (Aryal et al., 2012; Nawaz et al., 2014; Wu, 2014), have been degraded due to overgrazing in the Sanjiangyuan region (Li, Brierley, Shi, Xie, & Sun, 2012; Zhou, Zhao, Tang, Gu, & Zhou, 2005). More recent implementation of conservation measures in national parks by the Chinese government has resulted in better protection for its wildlife and their habitats (Dai, Li, et al., 2019). Yet, climate change remains of great concern, as it may negate current conservation efforts, including those set to protect Tibetan brown bears (Balzotti et al., 2016; Stephens et al., 2017; Su et al., 2018). Assessing climatically suitable habitat is a key step in developing proactive strategies that reduce the impacts of climate change on the brown bear.

### 4.1 Habitat analysis in current and future climate scenarios

The Himalaya region encompasses significant habitats for the brown bear (Aryal et al., 2012). However, the distribution range and suitable habitat area of brown bears in the Himalaya region have changed significantly since the 1990s. These changes were primarily caused by habitat fragmentation and loss (Nawaz et al., 2014). At present, there are large areas of suitable habitat distribution of brown bears in the southeastern region of Yangtze River Zone and the northern region of Yellow River Zone and northwestern region of Lancang River Zone. However, there were few suitable habitat areas in the western region of Yangtze River Zone. These results may be explained by differing key factors that affect species distribution at varying spatial scales, including temperature seasonality, precipitation seasonality, temperature constancy, and altitude (Su et al., 2018).

The range of potential suitable habitat will significantly reduce under the future climate scenario in most areas where brown bears are currently distributed, with no habitat available in the Lancang River Zone at all. Loss of suitable brown bear habitat due to climate change has been observed in modeling studies from other Asian countries. For example, a large proportion of potential suitable habitat for brown bear was predicted to be lost in India, Pakistan, and Nepal by 2050, with loss in protected areas anticipated to be severe (Su et al., 2018). Possible outcomes which could result without adequate conservation action include brown bear population crashes due to insufficient habitat and movement into residential areas would cause conflicts between humans and brown bears (Dai, Xue, Cheng, et al., 2019).

### 4.2 The altitude range of climate refugia and its typical land use types

Tibetan brown bears are adapted to high altitudes and can be found above 5,000 m (Wu, 2014). We found the climate refugia of brown bears had an altitude range of 4,307–5,524 m, with over half of climate refugia located between 4,300 and 4,600 m. Tibetan brown bears occupy a large elevational gradient corresponding with daily activity budgets. For examples, brown bears usually move to higher altitudes in the morning (before 10 hr) looking for rocks or dens to rest, and then descend to lower altitudes in the afternoon (after 18 hr) to forage (Wu, 2014).

Rocky outcrops along mountainsides provide brown bears areas of refuge for rest and concealment in the form of natural dens, while alpine steppe and alpine meadow house food sources for brown bears, such as marmots (*Marmota himalayana*) and pikas (*Ochotona curzoniae*). Hence, this type of land use is essential for the survival of brown bears and therefore needs to be greatly protected.

### 4.3 Potential migration paths for brown bears

We simulated potential migration routes for brown bears based on Circuit modeling and found that low‐resistance areas were primarily divided into three isolated parts under the current climate scenario: the southeastern region of the Yangtze River Zone, central region of the Lancang River Zone, and north central region of the Yellow River Zone (Figure 7a). Despite a low‐resistance region within the Lancang River Zone, brown bears movement to the Yangtze River Zone would be difficult, as the current was low at the border between the Yangtze River Zone and the Lancang River Zone. This may hinder gene flow and dispersal between these populations, ultimately reducing genetic diversity and decreasing species adaptability. The Yellow River Zone was found to be a high‐current area; nonetheless, because of the Yellow River Zone being geographically distant from the other two zones, along with absence of protected areas to serve as stepping stones, connectivity between brown bear populations in the Yangtze and Lancang River Zones is restricted.

Species ability to track suitable habitat in the future strongly depends on population dynamics and dispersal processes that evolve over time (Early & Sax, 2011). Current high‐current regions would dramatically decrease by the 2050s, with potential movement routes substantially shrinking. Emerging high‐current regions in the western area of the Yangtze River Zone would be relatively remote from current brown bear populations and hence would likely not facilitate adequate movement to new habitat. More meaningful regions for brown bear migration will depend on the persistence of high‐current routes in climate refugia.

### 4.4 Conservation implications

#### 4.4.1 Protecting climatically suitable habitat

Habitat quality and loss directly affect how wild animals exploit the resources available to them (Hiller, Belant, & Beringer, 2015). Loss of brown bear habitat causes shortages in natural food availability, which may increase dependence on food linked to anthropogenic sources, increasing levels of livestock depredation and human‐bear conflicts (Dai, Xue, Cheng, et al., 2019; Dai, Xue, Zhang, & Li, 2019; Su et al., 2018). Currently, suitable areas should be strictly protected to avoid loss of habitat and natural food sources with priority given to localities less susceptible to climate change. In these areas, it is paramount that the grassland is preserved via reduction of livestock grazing intensity. Compensation programs aimed at sustainable grazing practices may be an adequate solution for encouraging local communities to play a more active role in conserving their environment and complying with government regulations while still maintaining financial livelihoods.

#### 4.4.2 Establishing potential corridors

Choosing appropriate regions to establish ecological corridors between isolated habitat patches would be one of the most effective techniques for facilitating dispersal between brown bear populations (Ramiadantsoa, Ovaskainen, Rybicki, & Hanski, 2015). These corridors can also serve to increase habitat area. In 2012, Aryal et al. used an ecological corridor model to connect brown bears inside and outside of protected areas in Nepal to assist bear populations in adapting to anticipated climate patterns. Similarly, we designed potential corridors to connect brown bear populations in different zones of Sanjiangyuan National Park according to the climate refugia and potential movement paths, which could facilitate dispersal and gene flow of brown bears.

#### 4.4.3 Restructuring conservation areas

Large areas of suitable brown bear habitat were identified outside the national park under the future climate scenario. Brown bear populations may greatly benefit from inclusion of these areas via restructuring of Sanjiangyuan National Park boundaries. Further, because the Yellow River Zone is distant from the other two zones, a new protection area to serve as a stepping stone should be established to promote connectivity between populations in the Yangtze River Zone and Lancang River Zone. In addition, targeted management on the periphery of existing national park boundaries would be useful as an increase in human activity along habitat edges may prevent brown bear dispersal, thus reducing gene flow.

#### 4.4.4 Strengthening monitoring on brown bears

Most master plans for protected areas only address strategies to combat the early impact stages of climate change (Xu et al., 2017). It is not possible to holistically understand how wildlife will respond to climate change and what management strategies would be most effective (Li, Li, Xue, et al., 2018). Therefore, long‐term scientific standardized monitoring should be implemented in Sanjiangyuan National Park to regularly assess changes in the population status and habitat of brown bears. Action plans can then be developed as changes materialize, providing timely and continuous efforts to preserve this at‐risk species.

## 5 CONCLUSION

This study identified shifts in suitable habitat for Tibetan brown bears and the most important areas for connecting current and future habitat in the context of climate change. The Tibetan brown bear serves as an umbrella species, with its protection serving as a benefit for other sympatric wildlife. Applying this method to such species with relevant ecological information enables conservation biologists to develop precise climate‐landscape conservation plans. Determining refugia and climate connectivity enable the identification of the most efficient regions to maintain brown bear populations and strengthen habitat connectivity.

## CONFLICT OF INTEREST

None declared.

## AUTHOR CONTRIBUTIONS

Diqiang Li and Yadong Xue developed concept and led manuscript production. Yunchuan Dai performed the structure of manuscript and drafted the first version of the manuscript. Charlotte E. Hacker, Yuguang Zhang, and Wenwen Li led species distribution modeling and contributed to manuscript writing. Yu Zhang, Haodong Liu, Jingjie Zhang, and Yunrui Ji performed the data collection and data analysis. All coauthors participated in the scientific discussions and commented on the manuscript.

### OPEN RESEARCH BADGES

This article has been awarded https://openscience.com and https://openscience.com. All materials and data are publicly accessible via the Open Science Framework at http://www.worldclim.org/; https://doi.org/10.1002/ece3.3994; http://www.gscloud.cn/; https://doi.org/10.7717/peerj.3477; http://sedac.ciesin.columbia.edu/; https://doi.org/10.7717/peerj.3477.

## Data Availability

We used open‐access data from WorldClim (http://www.worldclim.org/; https://doi.org/10.1002/ece3.3994), ASTER GDEM V2 (http://www.gscloud.cn/; https://doi.org/10.7717/peerj.3477) and Last of the Wild, v2 (http://sedac.ciesin.columbia.edu/; https://doi.org/10.7717/peerj.3477).

## APPENDIX 1

### Correlation coefficient of environmental variables

**Table:**

Layer	Bio1	Bio2	Bio3	Bio4	Bio5	Bio6	Bio7	Bio8	Bio9	Bio10	Bio11	Bio12	Bio13	Bio14	Bio15	Bio16	Bio17	Bio18	Bio19	Altitude	HII
Bio1	1.0000																				
Bio2	0.3202	1.0000																			
Bio3	−0.0802	0.3298	1.0000																		
Bio4	0.1647	0.2491	−0.7984	1.0000																	
Bio5	0.9500	0.4111	−0.2752	0.4494	1.0000																
Bio6	0.9632	0.2383	0.0923	−0.0436	0.8653	1.0000															
Bio7	0.3622	0.4376	−0.6889	0.9566	0.6151	0.1370	1.0000														
Bio8	0.9701	0.3701	−0.2563	0.3957	0.9959	0.8909	0.5667	1.0000													
Bio9	0.9559	0.2731	0.1529	−0.1015	0.8355	0.9841	0.1031	0.8670	1.0000												
Bio10	0.9701	0.3700	−0.2563	0.3956	0.9959	0.8909	0.5666	1.0000	0.8670	1.0000											
Bio11	0.9682	0.2782	0.1332	−0.0824	0.8504	0.9886	0.1255	0.8825	0.9926	0.8825	1.0000										
Bio12	0.1482	−0.2964	0.5374	−0.8415	−0.1578	0.2674	−0.7323	−0.0811	0.3503	−0.0811	0.3429	1.0000									
Bio13	−0.0043	−0.2639	0.6377	−0.8918	−0.3023	0.1369	−0.8127	−0.2300	0.2214	−0.2301	0.2089	0.9683	1.0000								
Bio14	0.3424	−0.5108	0.0296	−0.4548	0.1212	0.4005	−0.3904	0.1917	0.4412	0.1917	0.4379	0.7667	0.6524	1.0000							
Bio15	−0.6277	0.2380	0.2632	0.0290	−0.5030	−0.5643	−0.1064	−0.5542	−0.5906	−0.5540	−0.6122	−0.4640	−0.2554	−0.7535	1.0000						
Bio16	0.0673	−0.2875	0.5961	−0.8785	−0.2374	0.2019	−0.7868	−0.1622	0.2851	−0.1622	0.2749	0.9896	0.9931	0.7091	−0.3495	1.0000					
Bio17	0.3440	−0.4584	0.1092	−0.5143	0.1062	0.4050	−0.4271	0.1764	0.4567	0.1764	0.4512	0.8320	0.7162	0.9619	−0.7621	0.7711	1.0000				
Bio18	0.0673	−0.2875	0.5961	−0.8785	−0.2374	0.2019	−0.7868	−0.1622	0.2851	−0.1622	0.2749	0.9896	0.9931	0.7091	−0.3495	1.0000	0.7711	1.0000			
Bio19	0.3235	−0.4632	0.0942	−0.4891	0.0987	0.3893	−0.4173	0.1656	0.4335	0.1655	0.4269	0.8011	0.7008	0.9446	−0.7271	0.7475	0.9865	0.7475	1.0000		
Altitude	−0.9040	−0.3138	0.3967	−0.4932	−0.9536	−0.7816	−0.6551	−0.9538	−0.7637	−0.9538	−0.7813	0.1333	0.3153	−0.1732	0.6247	0.2368	−0.1710	0.2368	−0.1521	1.0000	
HII	0.4698	0.1017	0.2990	−0.3668	0.2974	0.4963	−0.1930	0.3374	0.5479	0.3373	0.5535	0.5453	0.4455	0.4315	−0.4957	0.4960	0.4942	0.4960	0.4483	−0.3312	1.0000
